# Insight into antimicrobial resistance at a new beef cattle feedlot in western Canada

**DOI:** 10.1128/msphere.00317-23

**Published:** 2023-10-19

**Authors:** Daniel Kos, Brittany Schreiner, Stuart Thiessen, Tim McAllister, Murray Jelinski, Antonio Ruzzini

**Affiliations:** 1 Department of Large Animal Clinical Sciences, Western College of Veterinary Medicine, University of Saskatchewan, Saskatoon, Saskatchewan, Canada; 2 Namaka Farms, Inc., Outlook, Saskatchewan, Canada; 3 Lethbridge Research and Development Centre, Agriculture and Agri-Food Canada, Lethbridge, Alberta, Canada; 4 Department of Veterinary Microbiology, Western College of Veterinary Medicine, University of Saskatchewan, Saskatoon, Saskatchewan, Canada; 5 Department of Biochemistry, Microbiology and Immunology, College of Medicine, University of Saskatchewan, Saskatoon, Saskatchewan, Canada; University of Wisconsin-Madison, Madison, Wisconsin, USA

**Keywords:** antimicrobial resistance, water, waterborne bacteria, beef cattle production, surveillance study

## Abstract

In North America, beef production relies on the administration of antimicrobials to manage disease. Bovine respiratory disease (BRD) is the most significant disease of beef cattle, and antimicrobial resistance (AMR) to conventional therapies presents an existential risk to animal welfare and food production. While AMR surveillance programs are poised to help facilitate antimicrobial stewardship and decision making at feedlots, monitoring strategies for large numbers of animals at an individual or group level are time consuming and costly. Accordingly, we completed a pilot investigation of feedlot water bowls, which is an understudied interface between cattle and bacteria. By performing cultivation-dependent and cultivation-independent studies, we demonstrate that water bowl-dwelling bacteria can act as sentinel organisms for clinically relevant antimicrobial resistance genes (ARGs) and that cattle have an impact on the microbial communities in the bowls. Moreover, by sampling water at a feedlot site before animal arrival, we detected resistance to two antibiotics: florfenicol and tulathromycin. After just 4 weeks of operation, multidrug-resistant bacteria were routinely found in most water bowls. A comparison of ARGs encoded by five water bowl bacterial isolates along with previously reported source and wastewater metagenomes to those found in BRD pathogens confirmed the utility of using water samples for AMR surveillance.

**IMPORTANCE:**

A better understanding of how environmental reservoirs of ARGs in the feedlot relate to those found in animal pathogens will help inform and improve disease management, treatment strategies, and outcomes. Monitoring individual cattle or small groups is invasive, logistically challenging, expensive, and unlikely to gain adoption by the beef cattle industry. Wastewater surveillance has become standard in public health studies and has inspired similar work to better our understanding of AMR in feedlots. We derived our insights from sampling water bowls in a newly established feedlot: a unique opportunity to observe AMR prior to animal arrival and to monitor its development over 2 months. Importantly, the bacterial community of a single water bowl can be influenced by direct contact with hundreds of animals. Our results suggest that water bowl microbiomes are economical and pragmatic sentinels for monitoring relevant AMR mechanisms.

## INTRODUCTION

Beef production in North America consists of a network of cow-calf operations, markets, feedlots, and packing plants. Generally, a relatively large number of cow-calf operations supply calves to feedlots either directly or through an auction process wherein animals are shipped to central auction sites and then sold to feedlots in assembled groups based on traits such as weight and sex. Additional commingling occurs at the feedlot where cattle from multiple auctions converge and may undergo additional sorting. The stress of shipping and commingling has been linked to higher incidences of disease within the first weeks of arrival to the feedlot. Specifically, animals are prone to bovine respiratory disease (BRD), colloquially referred to as shipping fever, which is a multifactorial disease complex associated with stress from shipping, commingling, weather, and processing of the animals upon arrival (1). These mixing, sorting, and resorting increase the exposure of individuals to a constellation of viral and opportunistic bacterial pathogens. Accordingly, BRD, which is the most common cause of morbidity and mortality in feedlot cattle, is managed through vaccination, antimicrobial metaphylaxis, and treatment (2, 3).

In Canada, β-lactams, phenicols, macrolides, tetracyclines, and, as a last resort, fluoroquinolones are used to treat feedlot cattle (4, 5). Additional antibiotics such as virginiamycin—a mixed dose of a streptogramin and cyclic depsipeptide—are also approved for use but are not independently reported by national AMR surveillance programs (4). Quinolones and macrolides are both considered category I antimicrobials by the WHO, whereas Health Canada places quinolones in category I, and macrolides, in category II (6, 7). Metaphylaxis by macrolides is common practice in North America and has been suggested to be the most effective antimicrobial class to mitigate losses due to BRD (2). Nevertheless, the effective use of antimicrobials relies on an understanding of antimicrobial resistant determinants that accumulate in relevant pathogens and the respective reservoirs that contribute to their dissemination. In the context of agriculture, this includes both animal-associated and environmental reservoirs, including everything in between. The deposition of cattle manure within pens over decades can result in the accumulation of antimicrobial resistance genes (ARGs) (8), and there is evidence that selection for feedlot-specific ARGs occurs as the diversity of the resistome decreases and aligns with the types of antimicrobials administered upon entry into the feedlot (9). Thus, while antimicrobial use provides positive selective pressure for resistant organisms, the sources, transmission, and identities of ARGs at feedlots remain an active field of study.

Reliance on the widespread use of antimicrobials has resulted in the dissemination of ARGs throughout the biosphere. Environmental reservoirs of ARGs are ubiquitous and challenge the utility of antibiotics. Determinants that confer resistance to multiple drug classes and those that provide resistance to both human and veterinary pharmaceuticals represent existential threats to healthcare systems and agricultural food production. Disease and AMR surveillance using wastewater sampling is now a conventional approach that can be adapted to a variety of related research questions. In fact, water sources and effluents have been studied at feedlots to monitor AMR (9–12). Moreover, water has been associated with disease, specifically BRD, in both feedlot and dairy environments (13, 14). In one case, shared water troughs between pens were the most significant risk factor for BRD (14), although direct surveillance of microbes and ARGs is still lacking from this feedlot niche. To our knowledge, the bacterial communities that reside in water bowls have yet to be systematically characterized and, therefore, represent an understudied reservoir of AMR at the interface between the water source and animal.

In the fall of 2021, we were presented with a unique opportunity to study the microbiota present in the water bowls and changes to resident populations as cattle entered a new feedlot in western Canada. We rationalized that targeting the water bowl would also offer a pragmatic and non-disruptive sampling strategy for the feedlot industry to gain insight into microbial communities and potentially pathogen and AMR gene transmission. The results of applying both cultivation-dependent and cultivation-independent approaches to analyze the microbial communities in feedlot water bowls and their relationship to AMR observed in BRD pathogens are presented and discussed in the context of ongoing and future AMR surveillance platforms that will continue to inform antimicrobial use.

## RESULTS AND DISCUSSION

### Direct evaluation of aerobes living in water provides rapid insight into feedlot AMR status

**Fig 1:**
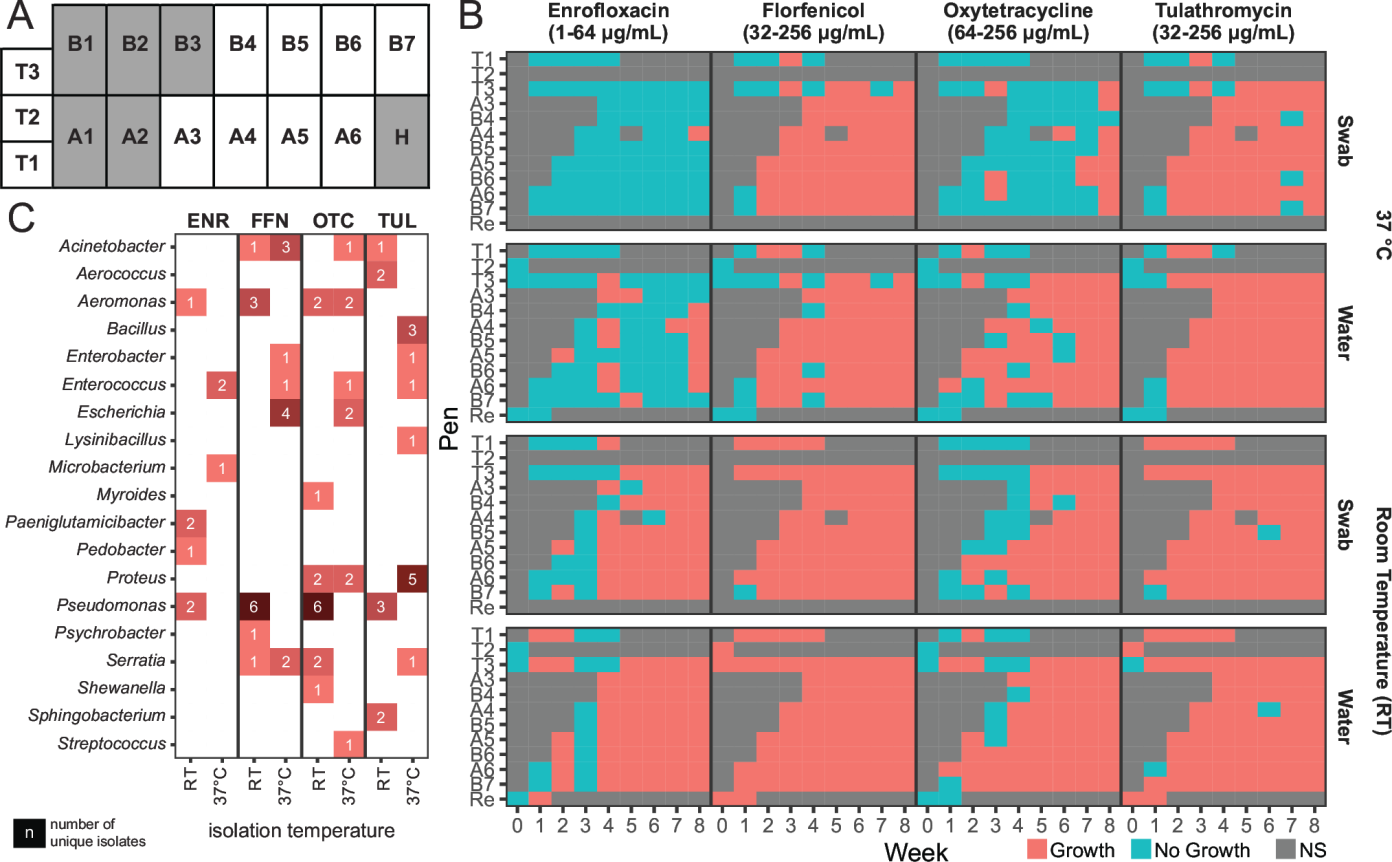
(A) A schematic overview of the feedlot. Individual pens are labeled, including home pens (A1-6; B1-7), transfer pens (T1-3), and a hospital pen (H). The pens that appear in white (A3-6, B4-7, and T1-3) were sampled during this study. (**B**) AMR profiles of water and swab samples taken from a feedlot for 8 weeks after animal arrival (week 0 indicates samples collected prior to animal arrival). The binary heatmap reports on the observation of growth (pink) or no growth (teal) in tryptic soy broth supplemented with antibiotics. Gray boxes are used for timepoints that were not sampled (NS) because animals had yet to be homed in these pens. (**C**) Heatmap showing the bacterial genera identified based on their isolation as morphologically distinct colonies obtained from water samples plated on different antibiotic-containing media incubated at RT and 37°C.

To overcome the limitations and impractical nature of individual and pooled cattle swabbing, we elected to sample and evaluate the antimicrobial susceptibility profiles of the animals’ water source. Furthermore, sampling prior to any animals arriving to the feedlot (Wk0) provided a rare opportunity to study the accumulation of ARGs in a feedlot. By collecting water samples and swabbing visible biofilms at the air-water interface, we were able to assess the presence or absence of aerobic AMR organisms. Four antimicrobials commonly used in feedlot cattle were selected for this experiment: enrofloxacin (ENR), tulathromycin (TUL), florfenicol (FFN), and oxytetracycline (OTC). Altogether, a total of 131 samples from 11 cattle pens over a 9-week period were collected (Fig. 1A; Table S1). Direct inoculation of water and biofilm samples into 96-well plates containing one of the four aforementioned antibiotics revealed the presence of AMR bacteria prior to populating the feedlot with cattle (Fig. 1B). In the Wk0 samples, we observed the growth of bacteria at room temperature (RT) in the presence of FFN and TUL at concentrations >32 µg/mL in samples from both the feedlot’s water reservoir and water bowls. Notably, AMR organisms were observed more routinely when experiments were conducted at RT compared to those at 37°C. The 37°C temperature was expected to restrict growth and was selected as it reflects body temperature as opposed to watering bowls, which were maintained at or above 10°C throughout the sampling period. Upon animal arrival, resistant bacteria, which we defined as those growing at >1 µg/mL ENR, >32 µg/mL FFN or TUL, and >64 µg/mL OTC, were observed within 4 weeks of animal arrival in both water and biofilms. A single exception was that OTC resistance took longer to develop in biofilm samples inoculated at 37°C than at RT. Nevertheless, it is reasonable to conclude that the impact of animals and feedlot practices, including the use of antimicrobials, may have contributed to the accumulation of AMR organisms in water bowls.

This data set demonstrated the recovery of organisms resistant to FFN and TUL prior to the entry of cattle or the use of antimicrobials at a newly established feedlot site. The data set also shows the recovery of AMR bacteria to all four antibiotics investigated occurred rapidly across the feedlot after animal entry and upon antimicrobial use. These antibiotics belong to four distinct classes: the fluoroquinolones (ENR), macrolides (TUL), phenicols (FFN), and tetracyclines (OTC). Pen-level observations of AMR occurred across all pens within 4 weeks of animal arrival. We suspect that both abiotic and biotic factors, including the dispersion of particulate matter (dust) across the site, shared fencing between adjacent pens, movement of animals within the feedlot, and the occurrence of disease and antimicrobial use throughout the feedlot, all contribute to the ability to recover AMR bacteria after 4 weeks.

### Antibiotic-resistant bacteria are readily isolated from water and bowl swabs

To begin to assign the bacterial sources of AMR in the water, we selected four pens for cultivation of bacteria on agar supplemented with the previously described four antimicrobials. Specifically, two pens used to temporarily house animals (pens T1 and T3) and two home pens (A6 and B7) were selected. We prioritized the isolation of bacteria from water samples rather than from biofilm swabs because water bowl type (plastic or stainless steel) varies across the industry in western Canada. A total of 13 water samples collected between weeks 1 and 8 resulted in the isolation of 75 distinct bacteria belonging to 19 genera (Fig. 1C; Tables S2 and S3). These bacteria were selected based on their ability to grow with either ENR, FFN, TUL, or OTC. Individual colonies were selected based on distinct morphologies, resulting in one to eight unique isolates per sample. Bacteria isolated from water samples incubated at 37°C and RT differed in that *Acinetobacter*, *Escherichia*, and *Enterococcus* were prevalent at 37°C, whereas *Pseudomonas* and *Psychrobacter* spp. dominated the isolates recovered from RT incubations.

**Fig 2:**
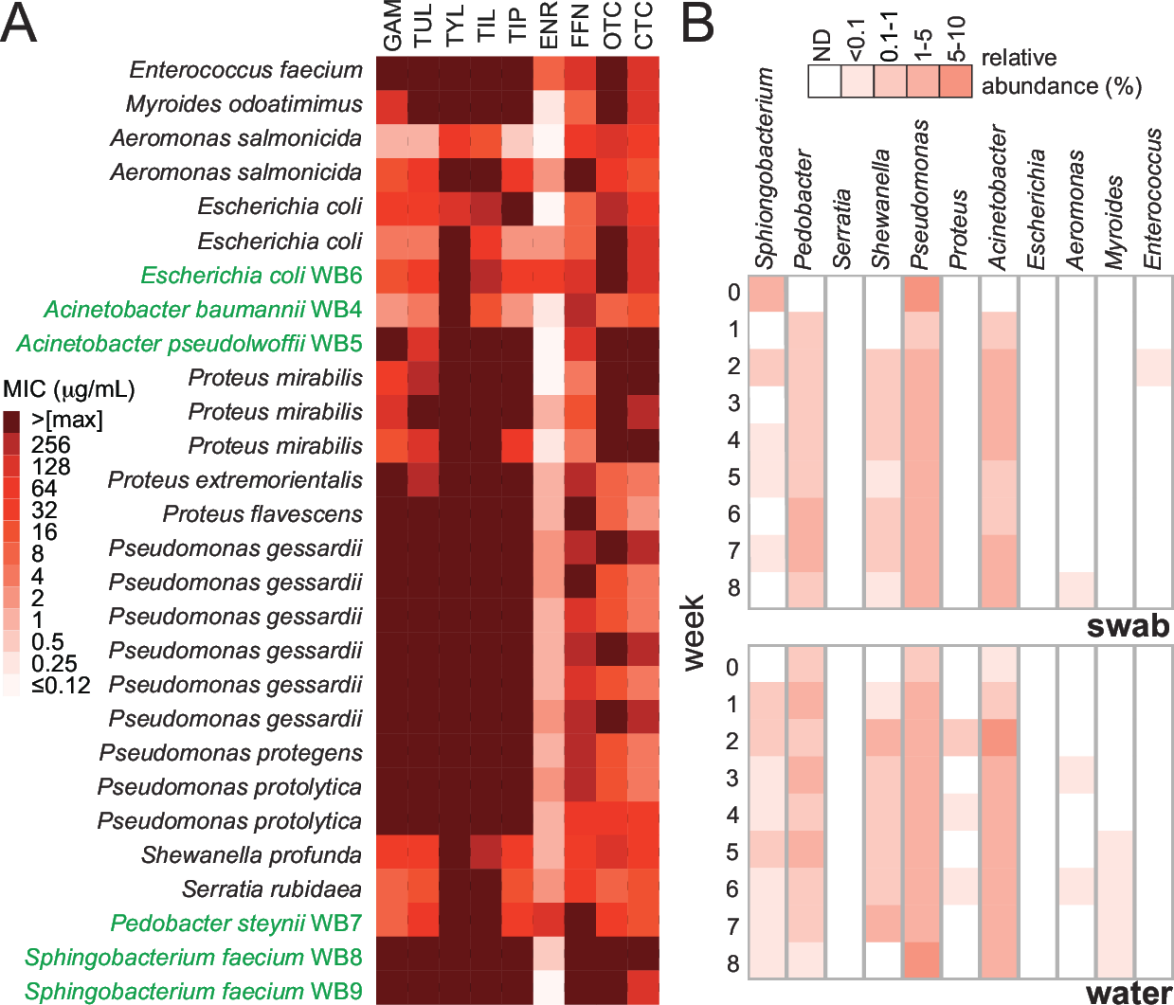
(A) Antimicrobial susceptibility profiles of 28 bacteria isolated from feedlot water bowls. Bacterial growth was evaluated in the presence 10 antimicrobials: the results from nine are shown, all isolated grew at 8 µg/mL penicillin, and six isolates (green, WB4-9) were further characterized by whole genome sequencing. (**B**) Heatmaps showing the relative abundance of isolated genera in cultivation-independent community profiles of water and swab samples before animal arrival at the feedlot and during the first 8 weeks of operation.

The isolation of bacteria based on resistance to a single antibiotic under aerobic conditions resulted in a collection of multidrug-resistant (MDR) organisms. In fact, further evaluation of the antimicrobial susceptibilities of the water bowl isolates to a panel of 10 drugs in five classes showed that a subset of the 75 isolates (28 in total) were resistant to at least three drug classes (Fig. 2A; Table S2), with resistance defined by MICs values >0.12 µg/mL for ENR, >16 µg/mL for tetracyclines, >32 µg/mL for FFN or macrolides, and >8 µg/mL for penicillin. The isolation of MDR bacteria was not unexpected. AMR is as ancient as antibiotic-producing bacteria (15), and AMR organisms have been found in pristine environments that have not been impacted by human antimicrobial use (16). The water in the feedlot bowls is sourced from an anthropogenically impacted freshwater system and is expected to contain AMR organisms. In fact, a recent metagenomic survey reported ARGs in the majority of ~350 lakes in Canada and listed *Acinetobacter* spp., a genus that we recovered from water using selection for three antibiotic classes, as a major contributor (17).

### AMR genera are represented in water bowl community profiles

**Fig 3:**
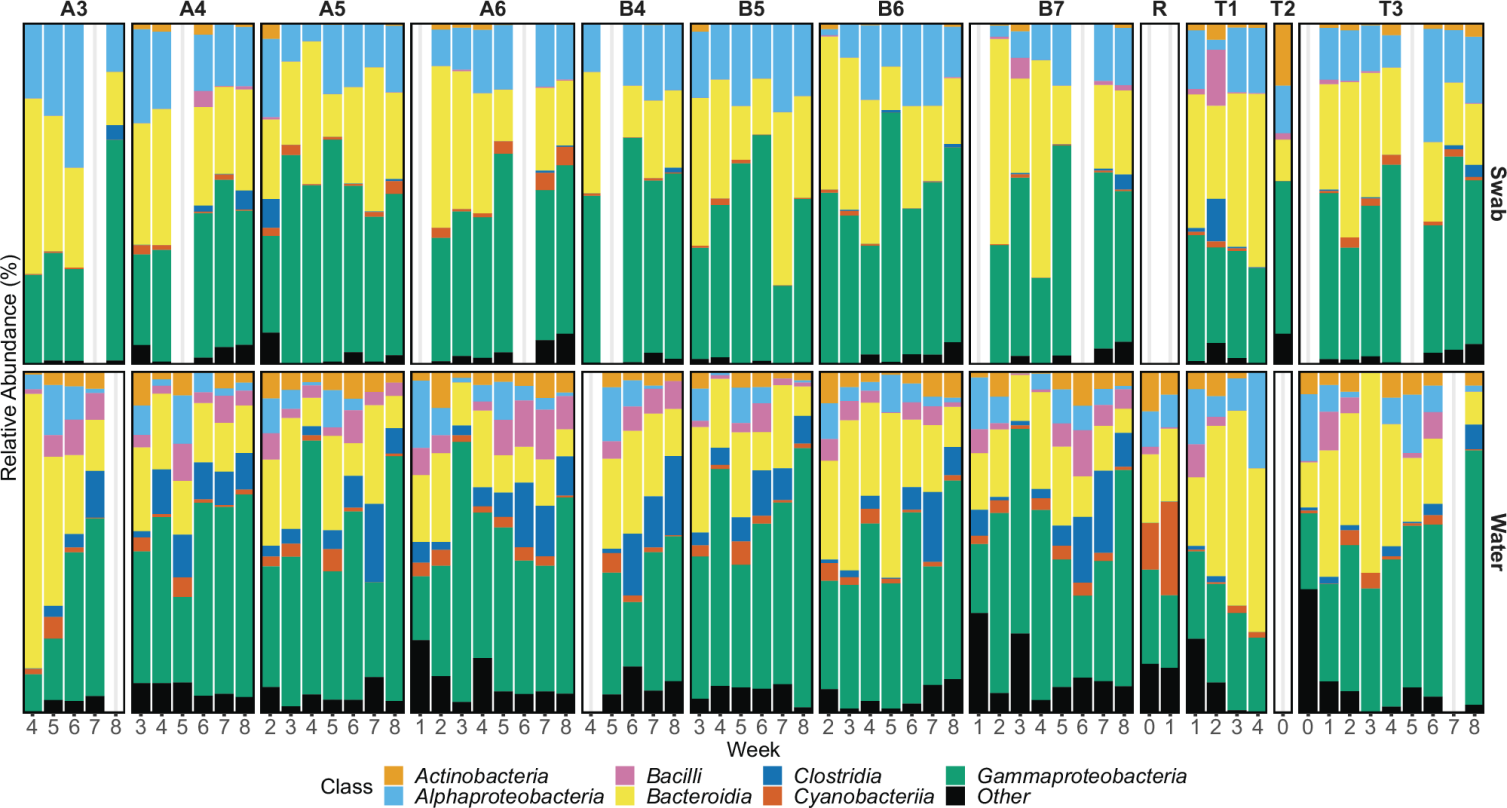
Seven most abundant bacterial classes present in water bowl swabs or water samples collected at a new feedlot. Pen IDs are provided at the top. Home pens (A3-A6 and B4-7) are separated from transfer (T1-3) pens by samples of the water source, a water reservoir (**R**) that was sampled before animal arrival and during the first week of operation. After animal arrival, the occupancy of pens T1-3 was variable (Table S1).

To evaluate how representative our biased collection of AMR aerobic bacterial isolates was to broader populations living in the water bowls, we performed microbial community profiling of both water and biofilms collected from the water bowls. The same samples that were used to monitor AMR were subjected to community profiling. Overall, several genera were well represented in both our 75-member isolate collection and the water communities. *Acinetobacter*, *Pseudomonas*, and *Pedobacter* were detected throughout the sampling period, whereas *Sphingobacterium* appeared sporadically based on amplicon sequencing data (Fig. 3B). It is noteworthy that two of these genera, *Acinetobacter* and *Pseudomonas*, are known to possesses intrinsic and acquired resistance mechanisms relevant to human and veterinary medicine, although their impact on the latter has not been as extensively studied (18). A member of the genus *Sphingobacterium* was recently shown to encode for a macrolide esterase that hydrolyzes tylosin (19), which was included in the animal diet during our study. In contrast to these ever-present genera, *Escherichia, Enterococcus*, and *Serratia* were not routinely detected in the community profiles (Fig. 2B), although these three genera were routinely isolated based on AMR phenotypes at 37°C. The selection bias applied to our cultivation-based methods may enrich for low abundance genera and/or those with a more transient existence in cold water. Nevertheless, these minor populations remain potential reservoirs of ARGs.

**Fig 4:**
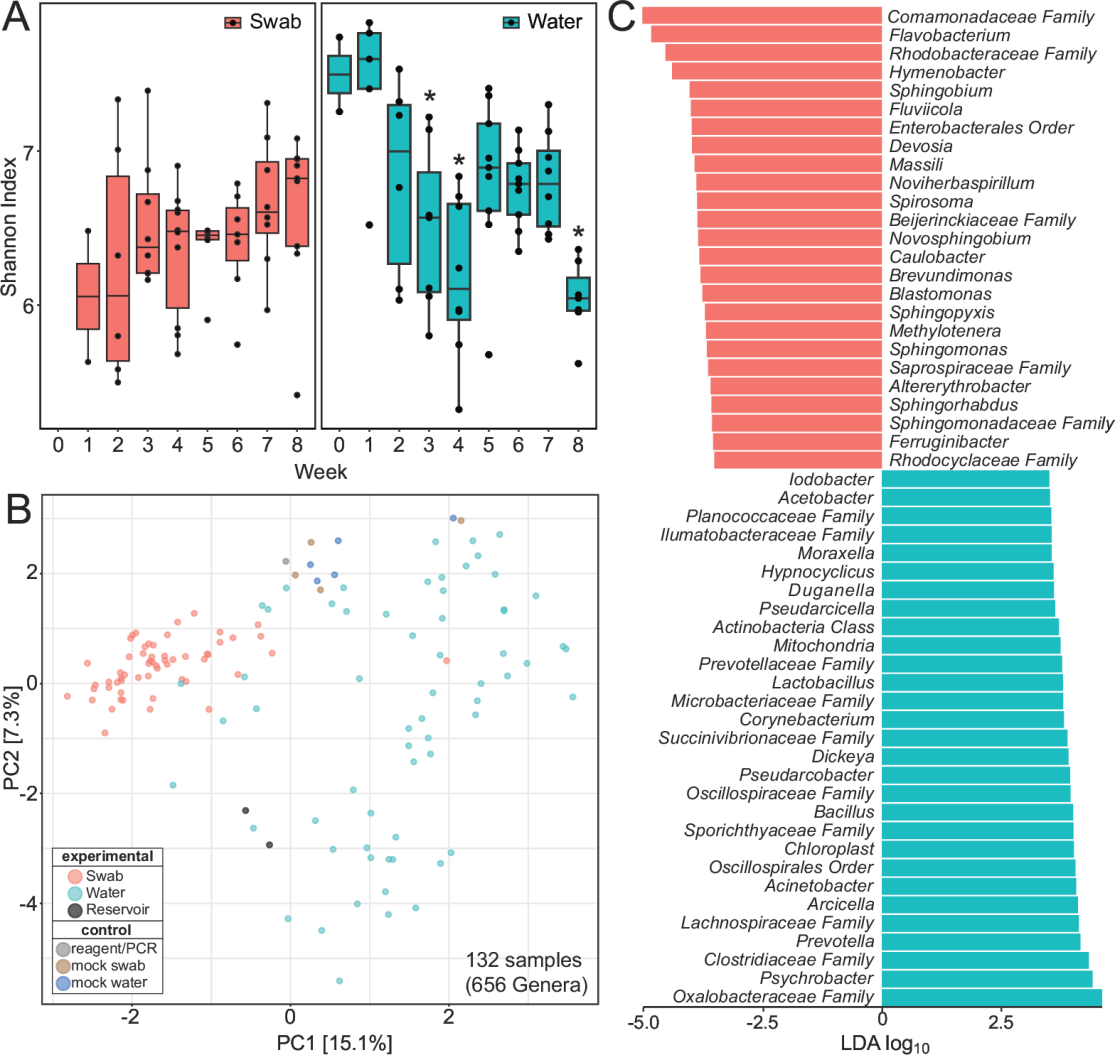
(A) Alpha-diversity of bacterial communities observed in swab and water samples represented weekly using Shannon diversity indices. The Shannon indices for swab communities did not differ significantly across samples. Significant differences between water samples collected before and after animal samples are indicated by asterisks (95% confidence, *P* < 0.08). (**B**) Beta-diversity of bacterial communities observed in swab and water samples, including analysis of the feedlot reservoir (the water source) and technical reagent and mock extraction controls. (**C**) Detected bacterial taxa that differ significantly in their abundances between water bowl swabs and water samples identified by linear discriminant analysis (LDA; >3.5 log_10_ taxa shown; *P* < 0.05).

The water and swab sample communities varied in their composition (Fig. 3; Fig. S2). At the level of class, water and the presumed biofilm communities were readily distinguished. Water was dominated by *Gammaproteobacteria* and *Bacteroidia*, whereas the biofilms also contained a comparatively high abundance of *Alphaproteobacteria*. Notably, a reduction in the relative abundance of *Cyanobacteria* was observed in the water bowls relative to the original water source, where it was the third most abundant taxon. This is likely a reflection of a differences between the biological niches: compared to the open reservoir, the water bowls are in direct contact with cattle and contain additional nutrients through the deposition of feed. Both animals and feed are likely to contribute growth substrates that would support the resident bacterial populations within water bowls. The overall alpha-diversity of the water and biofilm communities did not differ significantly during the study with a few exceptions (Fig. 4A). Reductions in diversity may be the consequence of antimicrobial use, as previously suggested (9, 20). A multitude of additional factors associated with feedlot practices that vary throughout the industry (e.g., animals and their health status, water bowl cleaning practices, feed) or feedlots location (e.g., climate) will also influence microbial diversity. For example, the ambient temperature became progressively colder over the course of the study (Table S1), although the water temperature was maintained in the bowls at ~10°C to avoid freezing.

Beta-diversity analysis clearly demonstrated significant differences in the bacterial communities associated with swab or water samples (Fig. 3B). Indeed, sample type variance (PERMANOVA: *F* = 20.8, *R^2^* = 0.13, *P* < 0.0001) was greater than that observed between collection weeks (*F* = 3.2, *R^2^* = 0.13, *P* < 0.0001) and pens (*F* = 1.8, *R^2^* = 0.10, *P* < 0.0001), which exhibited modest differences within sample types. Further inspection of bacterial communities by linear discriminant analysis (LDA) revealed differentially abundant taxa based on sample type (Fig. 3C). The influence of the animals was also apparent. In water, a relatively high abundance of *Moraxella* was observed. These bacteria are common residents of the upper respiratory tracts of cattle and account for ~50% of the bovine nasal microbiota (21), suggesting ready transfer of bacteria from the nasal cavity to water bowls. These bacteria are also closely related to *Psychrobacter* and *Acinetobacter*, which are widespread in nature and were also enriched in both water communities and our collection of AMR isolates. Finally, the *Microbacteriacea*e family, which includes members from the genera *Microbacterium* and *Paeniglutamicibacter*, were more abundant in water than in swab biofilm communities and were readily isolated from water using antibiotic selection, showing concordance between cultivation-independent and cultivation-dependent approaches.

### Water bowl isolates are rich in ARGs, including those of clinical relevance

While bacterial community profiles showed the influence of animals on water samples and revealed taxa that are notoriously associated with AMR (e.g*., Acinetobacter*), the plasticity of bacterial genomes precludes definitive predictions of ARGs within communities based on taxonomic identity. We explicitly investigated the AMR genotypes of six MDR isolates using long-read whole genome sequencing (Table S4). Two of the six isolates were *Acinetobacter* spp., which were selected based on their divergent AMR phenotypes and, more generally, due to their relative abundance in studied water communities. *Escherichia coli* and *Pedobacter steynii* were selected because they were either rarely (*E. coli*) or readily (*P. steynii*) detected in standard microbial community profiles and resistant to all five classes of antibiotics. Finally, two *Sphingobacterium faecium* isolates were selected based on this species’ aforementioned role in harboring feedlot-specific macrolide resistance to tylosin, tilmicosin, and tildipirosin (19). Although the genome of *Pedobacter steynii* WB7 does not encode for known ARGs, a homology-based informatic approach resulted in the identification of 40 distinct ARGs in the genomes of remaining five water bowl isolates (Table S5).

**Fig 5:**
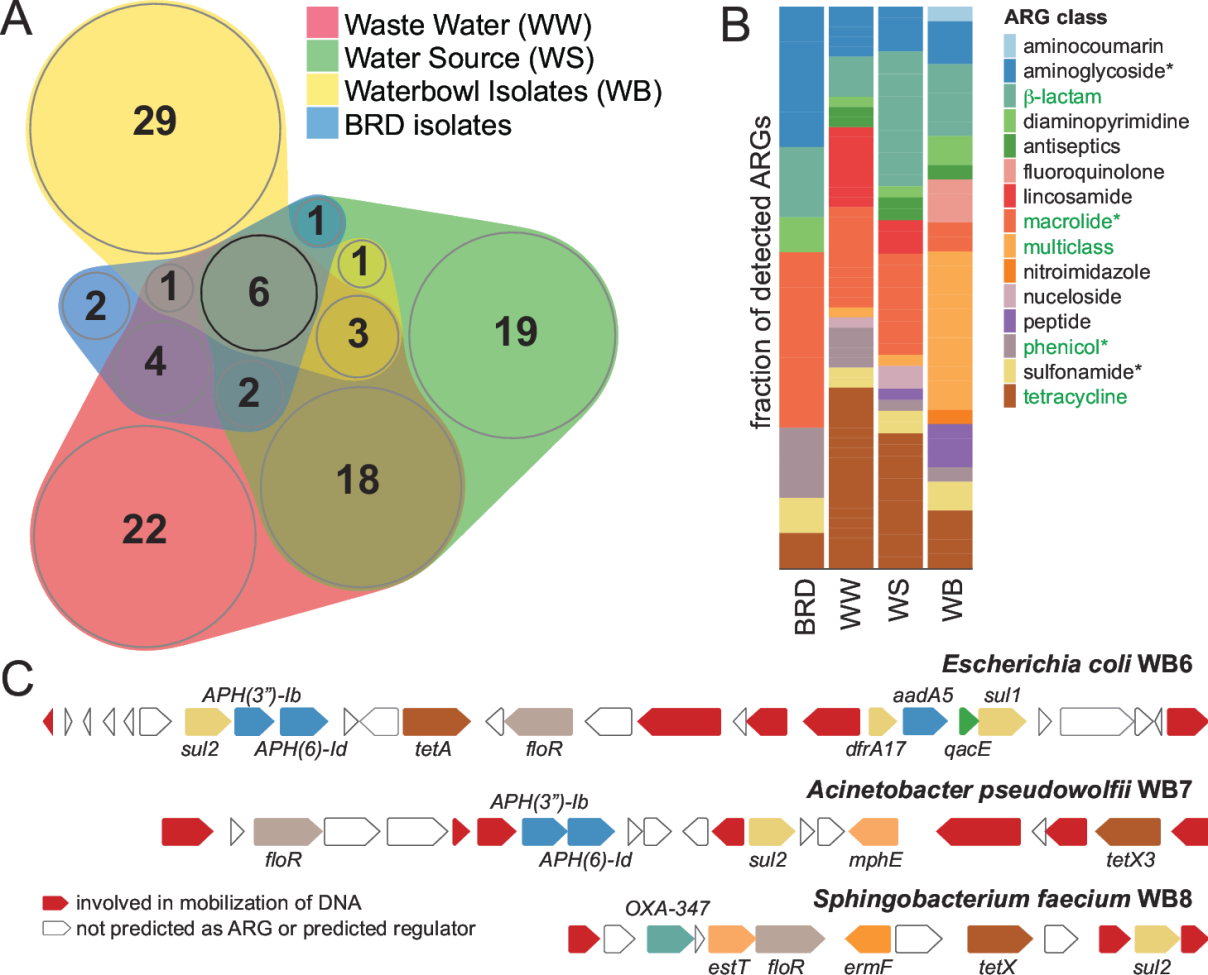
(A) Venn diagram showing ARGs that are commonly observed in distinct and overlapping environments. The ARGs identified in the whole genomes of five WB isolates were compared to the genomes of BRD pathogens (PRJNA281531, PRJNA306895, and PRJNA340884: 51 *P*. *multocida*, 98 *M*. *haemolytica*, and 23 *H*. *somni*), feedlot source water and wastewater metagenomes (PRJNA292471 and PRJNA529711). (**B**) Comparison of identified ARGs, by antibiotic class, across four sample types. Antibiotic classes listed in green were used at the feedlot during the study. Asterisks indicate that specific ARGs to these classes were observed in both water sample type metagenomes as well as within WB isolate and BRD pathogen genomes. (**C**) ARG clusters carried by MDR water bowl isolates. The *S. faecium* WB8 and WB9 genomes have syntenous ARG clusters (WB9 not shown). ARGs are colored coded by antibiotic class, and their identities are noted.

To begin to assess the relationship between these four bacterial genera and other feedlot-associated environmental reservoirs of AMR, we compared their single ARGs and multi-gene systems to those reported from metagenomic studies of water and the whole genomes of *Pasteurellaceae* that are involved in BRD (*Histophilus somni*, *Mannheimia haemolytica*, and *Pasteurella multocida*). This analysis revealed differences between sets of ARGs in distinct feedlot-associated niches (Fig. 5A and B); however, it also demonstrated that specific ARGs accumulate across environments. In particular, the ARGs observed in both WB isolates and BRD pathogens were found to be physically clustered or in the case of *S. faecium* WB8 and WB9 on an approximately 56-kb conjugative plasmid (Fig. 5C). A total of 6 ARGs (*aadA*, *APH(3”)-1b*, *APH (6)-Id*, *sul2*, *floR*, and *estT*) were found throughout water sample genomes and metagenomes as well as within a representative set of 172 BRD pathogens. Sulfonamides and aminoglycosides are not routinely used in feedlots (22) and were not used during the course of this study. Nevertheless, two aminoglycoside phosphotransferases and a sulfonamide resistant dihydropteroate synthase were observed in ARG clusters (Fig. 5C). In fact, the observation of ARGs shared between environments and across taxa is explained by their presence within gene clusters of mobile genetic elements. A central role for conjugative plasmids in the dissemination of ARGs across distinct sequence elements and organisms has been proposed (23). Thus, while the presence of *floR* (a phenicol exporter) and *estT* (a macrolide esterase) can be rationalized by the use of both classes of antibiotics during the study period, the current global status of the mobile resistome should not be ignored. ARGs are found throughout the biosphere with water systems, in particular, biological wastewater treatment facilities impacting their patterns of dissemination in mobile genetic elements of bacteria (24).

Our results suggest that antimicrobial use contributes to a niche, in this case within water bowls, which selects for MDR bacteria from the environment. Remarkably, the whole genome sequences of just 4 MDR isolates revealed 5/16 ARGs found in a much larger set of cattle pathogens. Furthermore, along with previously obtained water metagenomes, our limited data set was used to identify 14/16 ARGs in the same 3 *Pasteurellaceae* that contribute to BRD. Only two ARGs were unique to the pathogen genomes analyzed, *APH(3′)-1b* and *dfrA14*, although orthologs of the latter (e.g*., dfrA12* and *dfrA17*) were observed in distinct data sets. Thus, combined cultivation-dependent and cultivation-independent approaches using water for the isolation of both bacteria and DNA represent pragmatic approaches to gain insight into AMR at beef cattle feedlots. The water bowls, which are in direct contact with animals, appear to be an excellent proxy for AMR surveillance. Future studies of this sample type should be conducted to evaluate additional applications such as the detection and characterization of BRD pathogens.

## MATERIALS AND METHODS

### Facility and animals

A newly established cattle feedlot was the subject of our study. Mixed cross-bred beef calves or yearlings were administered tulathromycin (Draxxin, Zoetis, Kirkland, QC, Canada) 2.5 mg/kg body weight (BW) or oxytetracycline 20 mg/kg BW (Oxymycin LA 300, Zoetis) on arrival. Tylosin (Tylosin 40Bio Agri Mix, Mitchell, ON, Canada) was incorporated into the feed at a rate of 11 mg/kg/day. Cattle with clinical signs of BRD were administered one or more regimens of parenteral antibiotics: ceftiofur 6.6 mg/kg BW (Excede 200, Zoetis), florfenicol 40 mg/kg BW (Florkem Ceva Animal Health, Guelph ON, Canada), and marbofloxacin 10 mg/kg BW (Forcyl, Vetoquinol, Lavaltrie, QC, Canada). In general, cattle arriving at the feedlot were transited through receiving pens (T1-3) before allocation to home pens (A1-6 and B1-7: see Fig. 1A). Water was provided *ad libitum* via stainless-steel water bowls on an automated refilling system. Bowls were not emptied or cleaned during the collection period. Irrigation water was pumped to a reservoir, which supplied the watering bowls.

### Sample collection

Water and biofilm swabs were collected on Wk0, before the cattle arrived at the feedlot, and then on a weekly basis from 20 October 2021 (Wk1) until 9 December 2021 (Wk8; see Table S1). Water samples (~500 mL) were collected in commercial plastic water bottles from the water bowls and near the shoreline of the reservoir. Cotton swabs (BD 220144) were used to sample the water bowl biofilms; alternating sides of the bowl were sampled each week.

### Water and swab sample processing

Water samples were passed through two filters: an autoclaved coffee filter to remove large particulate materials followed by a 0.2-µm Nalgene Rapid-Flow Sterile Single Use Bottle Top Filter (Thermo Fisher Scientific). After filtration, the 0.2-µm filters were excised from their plastic cups, quartered, placed in 10 mL of M9 salt solution, and vortexed for 15 s. A 0.5-mL aliquot was used immediately for antimicrobial sensitivity testing (AST) and bacterial isolation, and the remaining 9.5-mL volume was centrifuged at 1,000 × *g* for 10 min, and resuspended in phosphate-buffered saline (PBS, pH 7.4) supplemented with 20% glycerol for cryopreservation at −80°C (Fig. S1). Swabs were processed by adding 3 mL of M9 salt solution to the transport unit, followed by vortexing for 10 s. Then, 250 µL was diluted and used immediately for AST and bacterial isolation while the remaining solution was centrifuged and stored as described above in PBS supplemented with 20% glycerol.

### AST and isolation of bacteria based on AMR

Water and swab-derived samples were used to inoculate 96-well microtiter plates containing a twofold dilution series of four antibiotics: enrofloxacin (ENR, 0.12 to 64 µg/mL), tulathromycin (TUL, 0.25 to 64 µg/mL), florfenicol (FFN, 0.25 to 64 µg/mL), and oxytetracycline (OTC, 0. 5 to 256 µg/mL). All AST experiments were performed in tryptic soy broth (TSB) amended with 150 µg/mL cycloheximide to suppress fungal growth. All antibiotics were obtained as high-purity (>95%) powders from scientific research vendors. The AST plates were incubated at either RT or 37°C overnight to 4 d, with growth evaluated daily. Samples were collected from wells with the highest growth-permitting concentrations of antibiotics, and serial dilutions thereof (10^−4^ to 10^−8^) were plated on TSB agar (TSA) containing a sub-inhibitory concentration of corresponding antibiotic for bacterial isolation. Well-isolated, morphologically distinct colonies were then sub-cultured in 5 mL of TSB for ~24 h before preparing 20% glycerol stocks. A total of 75 bacteria were taxonomically identified by comparing an approximately 400-bp region of their 16S rRNA gene to the NCBI 16S rRNA database. A subset of 28 isolates were subjected to additional AST using a panel of 10 antimicrobials that belong to five disparate classes that are commonly used in feedlot veterinary medicine as previously described (25).

### DNA isolation from water and swab samples and microbial community profiling

DNA was extracted and purified from water and biofilm swab samples utilizing GenElute bacterial genomic DNA (Sigma Aldrich) and Purelink Microbiome (Thermo Fisher Scientific) kits. A total of 122 samples, including controls, were subjected to bacterial community profiling according to the PCR amplified V3 and V4 regions of their 16S rRNA genes: the primer pair included indexing adaptors fused to the 341F (CCTACGGGNGGCWGCAG) and 805R (GACTACHVGGGTATCTAATCC) broad-range 16S rRNA gene-targeting primers (26). Sequencing was performed using the Illumina MiSeq PE300 platform at Genome Quebec (Montreal, QC, CA). The control samples and DNA extractions were performed on material processed as described above, including (i) the water that was emptied from the plastic bottles used for sample collection, (ii) unused sterile swabs, and (iii) a blank PCR amplification. Using QIIME2, sequence variants were generated by DADA2 and taxonomically sorted with a 16S rRNA pretrained classifier (27–30). Data visualization was performed using QIIME2, phyloseq, and microViz (31–33).

The Shannon diversity index for each sample was generated in QIIME2 with a sampling depth of 6,000, and an analysis of variance (ANOVA) and subsequent pairwise comparisons (Tukey honest significant differences) were performed on these data in R (4.2.2) (27–29, 31). After importing the taxonomic data into a phyloseq object using qiime2R, a PERMANOVA with Aitchison dissimilarity distance calculation (9,999 permutations), and principal component analysis (centered log ratio transformation) was generated from the genera of each sample with microViz in R (4.2.2) (31–33). An LDA effect size was performed on the 16S rRNA microbiome data using MicrobiomeMarker (34) in R (4.2.2). Samples were grouped by type (swab and water), read counts were normalized to counts per million, and the Kruskal–Wallis test (*P*-value <0.05) was employed.

### DNA isolation, whole genome sequencing, and analysis of AMR bacteria

DNA was isolated from six bacterial monocultures obtained from water bowls (WB, named WB4-9) grown in TSB using a Purelink Microbiome kit (Thermo Fisher Scientific). PacBio SMRT HiFi nucleotide sequencing and assembly (hifiasm) were performed at the University of Saskatchewan’s Global Institute for Food Security (35). Manual curation of the assembled data was performed to merge contigs with identical sequences. The detection of ARGs in assembled genomes and metagenomic data was performed using ABRicate (36) through comparison to protein sequences made available by the Comprehensive Antibiotic Resistance Database (April 2023) (37). In addition to the 6 WB genomes, the genomes of BRD-associated pathogens (*P. multocida n* = 51, *H. somni n* = 23, and *M. haemolytica n* = 98) were selected based on their isolation from cattle from NCBI BioProjects PRJNA281531, PRJNA306895, and PRJNA340884. Water metagenomic sequencing data were downloaded from PRJNA529711 and PRJNA292471 and assembled using SqueezeMeta v1.6.2, March 2023 using Megahit (38, 39). Short contigs (<200 bp) were removed using prinseq (40). Positive identifications of ARGs were determined by 80% identity and coverage thresholds. After automated gene identification, the list was manually curated to unify hits with redundant/synonymous nomenclature, count multicomponent systems as single entries, and remove regulatory proteins. A Venn diagram was then generated using nVennR in R (4.2.2) (41).

## Data Availability

The nucleotide sequences of bacteria characterized in this study are available through GenBank BioProjects PRJNA820789 (whole genome sequences) and PRJNA976740 (microbial community profiles).

## SUPPLEMENTAL MATERIAL

Supplemental Material msphere.00317-23-s0001.pdf Fig. S1, Fig. S2, and Tables S1 to S5.

ASM does not own the copyrights to Supplemental Material that may be linked to, or accessed through, an article. The authors have granted ASM a non-exclusive, world-wide license to publish the Supplemental Material files. Please contact the corresponding author directly for reuse.
