# Supplementary material for: Insight into antimicrobial resistance at a new beef cattle feedlot in western Canada
Source: mSphere. 2023 Oct 19;8(6):e00317-23. doi: 10.1128/msphere.00317-23 (PMC10732036; doi:10.1128/msphere.00317-23)
Supplement: Supplemental Material — Fig. S1, Fig. S2, and Tables S1 to S5. [file msphere.00317-23-s0001.pdf]

Supplemental Materials for

Insight into antimicrobial resistance at a new beef cattle feedlot in western Canada

Daniel Kos[1], Brittany Schreiner[1], Stuart Thiessen[2], Tim McAllister[3], Murray Jelinski[1*], and Antonio Ruzzini[4,5,*]

[1]Department of Large Animal Clinical Sciences, Western College of Veterinary Medicine, University of Saskatchewan, Saskatoon, SK, Canada, S7N 5B4

[2]Namaka Farms Inc, Outlook, SK, Canada, S0L 2N0

[3]Lethbridge Research and Development Centre, Agriculture and Agri-Food Canada, Lethbridge, AB, Canada, T1J 4B1

[4]Department of Veterinary Microbiology, Western College of Veterinary Medicine, University of Saskatchewan, Saskatoon, SK, Canada, S7N 5B4

[5]Department of Biochemistry, Microbiology and Immunology, College of Medicine, University of Saskatchewan, Saskatoon, SK, Canada, S7N 5E5

*to whom correspondence should be addressed

Table of Contents

Table S1. Summary of feedlot sampling S2
Figure S1. Workflow diagram of sample collection S2
Table S2. Summary of MICs measured for 28 water bowl-isolated bacteria and 10 antibiotics S3
Table S3. List of additional water bowl-dwelling bacteria isolated based on antibiotic resistance S4
Figure S2. Bacterial community profiles showing 20 most abundant genera S5
Table S4. Summary of WB isolate genome assemblies S6
Table S5. ARGs detected in 5 feedlot water bowl isolates S6

Table S1. Summary of feedlot sampling

				Pen ID*											
**Week**	**Date**	**Time**	**Temp**	**R**	**T3**	**T2**	**T1**	**A6**	**A5**	**A4**	**A3**	**B7**	**B6**	**B5**	**B4**
0	15/09/21	15:00	12°C	4											
0	12/10/21	11:00	-1°C		1$^{\alpha}$†	1$^{\alpha}$†									
1	20/10/21	10:00	-2°C	4	2		2	1†				1†			
2	27/10/21	9:00	1°C		2		2	2	2			2	2		
3	03/11/21	9:30	-3°C		2$^{\alpha}$		2$^{\alpha}$	2	2	2		2	2	2	
4	10/11/21	9:30	1°C		2$^{\alpha}$		2$^{\alpha}$	2	2	2	2	2	2	2	1†
5	17/11/21	11:00	-8°C		1†			2	2	1††	2	2	2	2	1†
6	24/11/21	9:30	-14°C		2			1†	2	2	2	1†	2	2	2
7	1/12/21	9:30	4°C		1†			2	2	2	1†	2	2	2	2
8	8/12/21	9:30	-5°C		2			2	2	2	1†	2	2	2	2

R: Feedlot reservoir, two separate 4 x 500 mL water samples were pooled for analysis annotated as Week 0 and 1.
T1-T3: transfer pens used to temporarily hold animals (these pens are half the size/half the occupancy as home pens)
A & B: home pens, holding capacity of ~350 animals/pen. Not sampled prior to animal arrival at the feedlot.
*values under the Pen ID heading refer to the number of sample collected: one water and one swab (2/week).
Filled grey boxes indicate that samples were not collected.
†: Not enough DNA yield from either a water or swab sample; ††: No swab sample collection due to ice
α: Cattle were not present in pen

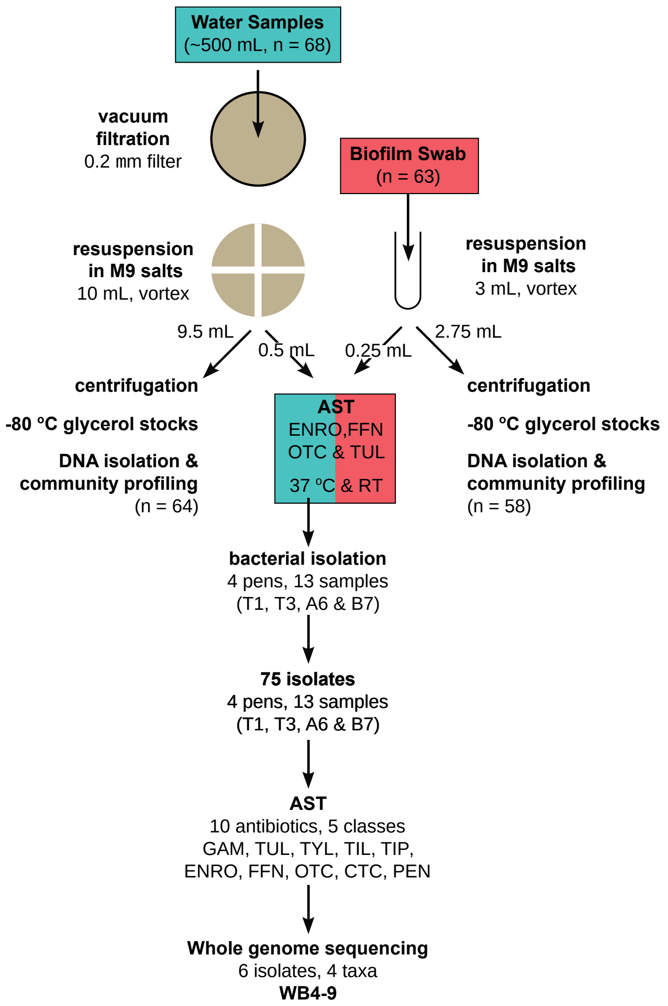

Figure S1. Workflow diagram of sample collection and processing of feedlot water bowl samples.

Table S2. Summary of 28 bacteria isolated from water bowls for which MICs were measured for 10 distinct antibiotics[a]

Isolation Conditions					MIC (μg/mL)									
**Pen**	**Week**	**Antibiotic & Temp**		**Taxonomic ID**[b]	**GAM**	**TUL**	**TYL**	**TIL**	**TIP**	**ENR**	**FFN**	**OTC**	**CTC**	**PEN**
T3	2	FFN	37 °C	*Acinetobacter baumannii* WB4	2	4	>128	16	2	0.25	256	8	16	>8
B7	5	FFN	RT	*Acinetobacter pseudolwoffii* WB5	>256	128	>128	>256	>128	≤0.12	128	>256	>256	>8
A6	5	FFN	37 °C	*Escherichia coli* WB6	16	32	>128	256	32	32	128	>256	128	>8
T3	8	ENR	RT	*Pedobacter steynii* WB7	8	64	>128	>256	32	128	>256	32	16	>8
B7	8	TUL	RT	*Sphingobacterium faecium* WB8	>256	>256	>128	>256	>128	≤0.12	>256	>256	128	>8
T3	6	TUL	RT	*Sphingobacterium faecium* WB9	>256	>256	>128	>256	>128	0.5	>256	>256	>256	>8
A6	8	FFN	RT	*Aeromonas salmonicida*	1	1	64	16	0.5	≤0.12	64	128	32	>8
A6	8	OTC	37 °C	*Aeromonas salmonicida*	16	64	>128	>256	64	2	>256	64	16	>8
A6	8	TUL	37 °C	*Enterococcus hirae*	>256	>256	>128	>256	>128	8	128	>256	128	>8
T3	1	OTC	37 °C	*Escherichia coli*	32	32	128	256	>128	≤0.12	8	256	64	>8
T3	2	OTC	37 °C	*Escherichia coli*	4	4	>128	64	2	2	8	>256	128	>8
T3	6	OTC	RT	*Myroides odoratimimus*	128	>256	>128	>256	>128	0.25	8	>256	128	>8
A6	1	OTC	37 °C	*Proteus mirabilis*	32	256	>128	>256	>128	≤0.12	4	>256	>256	>8
A6	5	TUL	37 °C	*Proteus mirabilis*	128	>256	>128	>256	>128	1	16	>256	256	>8
T3	6	TUL	37 °C	*Proteus mirabilis*	16	128	>128	>256	64	0.25	4	>256	>256	>8
A6	8	OTC	RT	*Pseudomonas gessardii*	>256	>256	>128	>256	>128	2	256	>256	256	>8
B7	8	OTC	RT	*Pseudomonas gessardii*	>256	>256	>128	>256	>128	2	256	>256	256	>8
T1	4	TUL	RT	*Pseudomonas gessardii*	>256	>256	>128	>256	>128	1	128	16	4	>8
T3	1	OTC	RT	*Pseudomonas gessardii*	>256	>256	>128	>256	>128	1	64	64	32	>8
T3	8	OTC	RT	*Pseudomonas gessardii*	>256	>256	>128	>256	>128	1	256	>256	256	>8
T3	6	FFN	RT	*Pseudomonas gessardii*	>256	>256	>128	>256	>128	2	>256	16	4	>8
T1	1	ENR	RT	*Pseudomonas proteolytica*	>256	>256	>128	>256	>128	2	256	16	4	>8
T3	1	ENR	RT	*Pseudomonas proteolytica*	>256	>256	>128	>256	>128	1	128	16	4	>8
T1	1	TUL	RT	*Pseudomonas extremorientalis*	>256	256	>128	>256	>128	1	256	8	4	>8
B7	5	TUL	RT	*Pseudomonas flavescens*	>256	>256	>128	>256	>128	1	>256	8	2	>8
A6	1	FFN	RT	*Pseudomonas protegens*	>256	>256	>128	>256	>128	1	256	16	4	>8
T3	1	FFN	37 °C	*Serratia rubindaea*	8	16	>128	>256	16	2	32	8	16	>8
T3	5	OTC	RT	*Shewanella profunda*	32	32	>128	256	32	1	32	128	32	>8

a – the isolates highlighted in green were prioritized for whole genome sequencing experiments

b – top hits are presented based on at least 400 bp of the 16S rRNA gene sequence

Table S3. Alphabetical list of 47 additional water bowl-associated bacteria isolated based on antibiotic resistance

Pen	Week	Isolation Conditions Antibiotic & Temp		Taxonomic ID[a]
T3	8	FFN	37 °C	*Acinetobacter baumannii*
T3	6	FFN	37 °C	*Acinetobacter baumannii*
T1	1	OTC	37 °C	*Acinetobacter indicus*
T3	2	TUL	RT	*Acinetobacter indicus*
B7	3	TUL	RT	*Aerococcus urinaeequi*
T3	2	TUL	RT	*Aerococcus viridans*
T3	8	FFN	RT	*Aeromonas enterica*
A6	8	OTC	RT	*Aeromonas rivipollensis*
A6	8	ENR	RT	*Aeromonas salmonicida*
B7	8	FFN	RT	*Aeromonas salmonicida*
B7	8	OTC	37 °C	*Aeromonas salmonicida*
T3	2	OTC	RT	*Aeromonas salmonicida*
B7	8	TUL	37 °C	*Bacillus haynesii*
T1	4	TUL	37 °C	*Bacillus paralicheniformis*
T1	4	TUL	37 °C	*Bacillus pumilus*
B7	1	FFN	37 °C	*Enterobacter hormaechei*
B7	1	TUL	37 °C	*Enterobacter cloacae*
B7	8	ENR	37 °C	*Enterococcus*
A6	8	OTC	37 °C	*Enterococcus hirae*
A6	8	ENR	37 °C	*Enterococcus hirae*
B7	1	FFN	37 °C	*Enterococcus mundtii*
A6	8	FFN	37 °C	*Escherichia coli*
T1	1	FFN	37 °C	*Escherichia coli*
T3	1	FFN	37 °C	*Escherichia coli*
B7	5	TUL	37 °C	*Lysinibacillus louembei*
B7	5	ENR	37 °C	*Microbacterium chocolatum*
B7	8	ENR	RT	*Paeniglutamicibacter antarcticus*
T3	2	ENR	RT	*Paeniglutamicibacter antarcticus*
A6	1	TUL	37 °C	*Proteus mirabilis*
T3	6	TUL	37 °C	*Proteus mirabilis*
A6	5	TUL	37 °C	*Proteus mirabilis*
B7	1	OTC	37 °C	*Proteus mirabilis*
B7	1	OTC	RT	*Proteus mirabilis*
T3	1	OTC	RT	*Proteus mirabilis*
T3	1	FFN	RT	*Pseudomonas edaphica*
T3	6	FFN	RT	*Pseudomonas fluorescens*
B7	1	FFN	RT	*Pseudomonas fulva*
T1	1	FFN	RT	*Pseudomonas gessardii*
T1	1	OTC	RT	*Pseudomonas gessardii*
T1	4	OTC	RT	*Pseudomonas gessardii*
A6	5	FFN	RT	*Psychrobacter maritimus*
B7	1	OTC	RT	*Serratia marcescens*
T1	4	FFN	RT	*Serratia marcescens*
B7	1	OTC	RT	*Serratia marcescens*
A6	1	FFN	37 °C	*Serratia rubidaea*
B7	1	TUL	37 °C	*Serratia rubidaea*
T3	5	OTC	37 °C	*Streptococcus equinus*

a – top hits are presented based on at least 400 bp of the 16S rRNA gene sequence

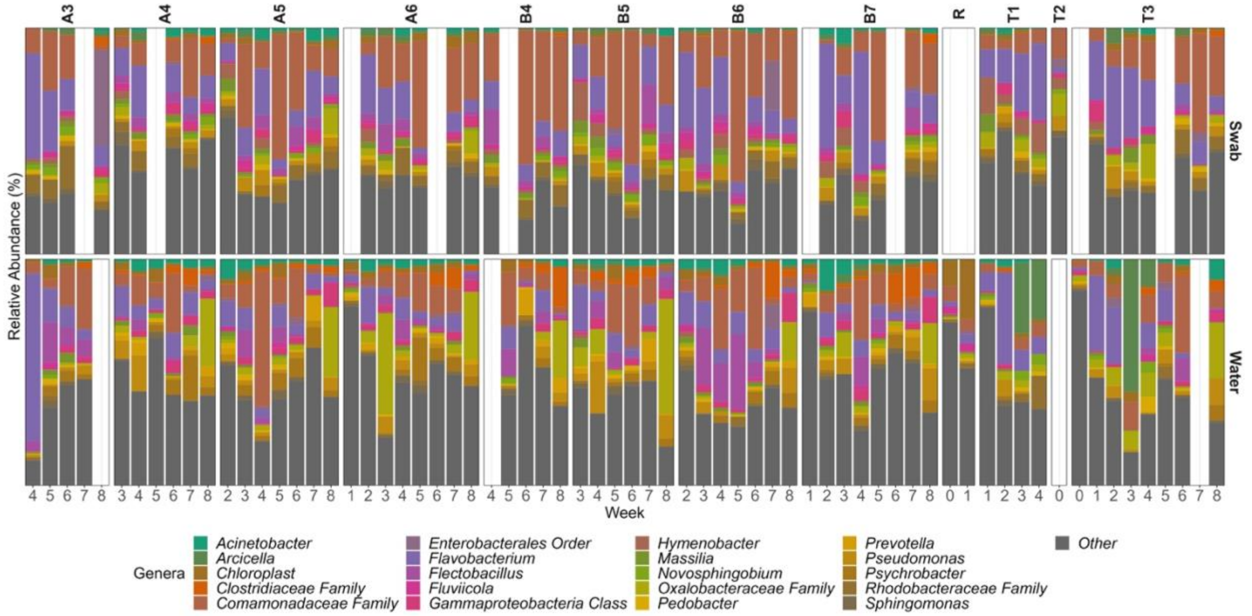

Figure S2. The composition of bacterial communities present in water bowl swabs or water samples collected at a new feedlot represented using the 20 most abundant genera. Pen IDs are provided at the top. Home pens (A3-A6 and B4-7) are separated from transfer (T1-3) pens by samples of the water source, a water reservoir (R) that was sampled before animal arrival and during the first week of operation.

Table S4. Summary of water bowl isolate bacterial genome assemblies

	replicon	Genbank ID	length	GC (%)	no. of ARGs*
***Acinetobacter baumanni* WB4**	chromosome	CP123854	3.80 Mb	39.0	9
***Acineobacter pseudolwoffi* WB5**	chromosome	JARXQN010000001	2.92 Mb	43.6	0
	plasmid	JARXQN010000002	163 kb	41.2	4
***Escherichia coli* WB6**	chromosome	JARXQO010000001	5.04 Mb	50.6	26
Pedobacter styenii	chromosome	CP123860	6.19 Mb	40.6	0
***Sphingobacterium faecium* WB8**	chromosome	CP123861	5.30 Mb	36.9	0
	plasmid	CP123862	55.7 kb	34.2	6
***Sphingobacterium faecium* WB9**	chromosome	JARXQP010000001	4.62 Mb	37.3	0
	plasmid	JARXQP010000002	55.9 kb	34.4	6

*ARG detection based on protein sequences in the CARD (April 2023) + EstT; multigene systems are counted once (*e.g. mdtABC* = 1 ARG entry)

Table S5. ARGs detected in 5 feedlot water bowl isolates using the CARD*

	Acinetobacter			***Sphingobacterium faecium***	
**Class**	***baumannii* WB4**	***pseudolwoffii* WB5**	***Escherichia coli* WB6**	**WB8**	**WB9**
**Aminocoumarin**			*mdtABC,*		
**Aminoglycoside**		*APH(3'')-Ib, APH(6)-Id*	*aadA, APH(6)-Id, kdpE*		
**β-lactam**	*ADC, OXA-695*		*EC-14, TEM*	*OXA-347*	*OXA-347*
**Diaminopyrimidine**			*dfrA12, dfrA17*		
**Disinfecting agents and antiseptics**			*qacEdelta1*		
**Fluoroquinolone**	*abaQ*		*emrA*		
**Macrolide**				*ermF, estT*	*ermF, estT*
**Multi-Class**	*abeS, abeM, adeFGH, adeIJK, AmvA*		*acrAB, acrEF, KpnEF, mdfA, mdtEF. mdtM, mdtNOP*		
**Nitroimidazole**			*msbA*		
**Peptide antibiotic**			*bacA, eptA, PmrF-ugd*		
**Phenicol**		*floR*	*floR*	*floR*	*floR*
**Sulfonamide**			*sul1, sul2*	*sul2*	*sul2*
**Tetracycline**	*adeABC*	*tet(X)*	*emrKY, tet(A)*	*tet(X)*	*tet(X)*
